# Reduced Graphene Oxide–Based Spectrally Selective Absorber with an Extremely Low Thermal Emittance and High Solar Absorptance

**DOI:** 10.1002/advs.201903125

**Published:** 2020-02-27

**Authors:** Qihua Liao, Panpan Zhang, Houze Yao, Huhu Cheng, Chun Li, Liangti Qu

**Affiliations:** ^1^ Key Laboratory for Advanced Materials Processing Technology Ministry of Education of China State Key Laboratory of Tribology Department of Mechanical Engineering Tsinghua University Beijing 100084 P. R. China; ^2^ Department of Chemistry Tsinghua University Beijing 100084 P. R. China; ^3^ School of Chemistry and Chemical Engineering Beijing Institute of Technology Beijing 100081 P. R. China

**Keywords:** graphene, solar energy conversion, solar steam escape, spectrally selective absorbers

## Abstract

Carbon‐based black materials exhibit strong solar absorptance (α_solar_ >0.90), which play key roles in transforming solar energy into available power for solar‐thermal, thermophotovoltaic, thermoelectric, and many other systems. However, because of high thermal emittance (>95%), these carbon‐based materials always cause huge energy loss that hinders the solar‐thermal conversion efficiency tremendously. In this study, a reduced graphene oxide–based spectrally selective absorber (rGO‐SSA) is demonstrated, which possesses a recorded low thermal emittance (≈4%) and high solar absorptance (α_solar_ ≈ 0.92) by easily regulating the reduction level of inner 2D graphene sheets. Compared to conventional carbon‐based black materials, thermal emittance of this rGO‐SSA is largely reduced by ≈95.8% and the cutoff wavelength of rGO‐SSA is broadband‐tunable that can range from 1.1 to 3.2 µm. More importantly, this simply sol‐gel coated rGO‐SSA has high temperature tolerance at 800 °C for 96 h that is hardly achieved by other cermet‐based or photonic‐based SSAs. Based on this rGO‐SSA, ultrafast solar steam escape (0.94 mg cm^−2^ s^−1^) under concentrated solar irradiance is achieved directly. The insight from this study will provide a new strategy for constructing thermally stable carbon‐based SSAs and greatly facilitate the solar‐thermal practical significance.

## Graphical abstract

Spectral selectivity of hundred nanometer graphene thin film is demonstrated, and a reduced graphene oxide–based spectrally selective absorber (rGO‐SSA) is developed, which possesses an extremely low thermal emittance (≈4%) and high solar absorptance (α_solar_ ≈ 0.92). This work provides a new strategy for constructing thermally stable carbon‐based SSAs and greatly facilitates the solar‐thermal practical significance.

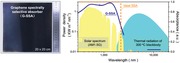

## 1 Introduction

The effective utilization of solar radiation has greatly boosted the burgeoning research fields including solar thermal,^1^ photovoltaic,^2^ thermophotovoltaic,^3^ thermoelectric,^4^ and many other systems.^5^ Among them, solar absorber with high solar radiation absorptance plays a key role in transforming solar energy into other sorts of available power. However, huge thermal radiation to environment of conventional solar absorbers always causes tremendous heat losses, restricting the solar thermal conversion efficiency. Spectrally selective absorbers (SSAs) have promising characters of high solar radiation absorptance and low thermal radiation emittance allowing them to achieve an efficient solar thermal conversion. Since proposed by Tabor at mid‐1950s, massive developments of material, structure, and fabrication method of low‐temperature (*T* < 100 °C) SSAs are reported^6^ and commercially used in domestic water heating systems. However, recently emerging mid‐ to high‐temperature systems address new challenges to constructing high temperature stable SSAs. For example, cermet‐based SSAs (TiNOX,^7^ W‐Ni‐YSZ,^8^ etc.), in which metal nanoparticles are imbedded in the dielectric matrix, have reached a high solar absorptance (α_solar_ ≈ 0.90) and low thermal emittance at 100 °C (ε_100_ ≈ 0.05, defined in Section S1, Supporting Information). However, the spontaneous diffusion of metal nanoparticles in cermet‐based SSAs at high temperatures (>600 °C) is inevitable,^9^ seriously weakening their spectral selectivity and durability. Photonic‐based SSAs (W‐PhC,[qv: 3a,b] Ni‐nanopyramids,^10^ etc.) with periodical nanostructures have enabled all‐metallic selective surfaces with excellent high‐temperature stability (>800 °C), nevertheless, their scale‐up ability is limited in laboratory level (several centimeters) for the complicated and expensive fabrication processes such as atomic layer deposition (ALD),[qv: 3a] lithographic,[qv: 3b,10] reactive ion etching (RIE),[qv: 3b] and so on.

Carbon‐based materials, such as carbon nanotubes, fullerene, amorphous carbon, and so on, have attracted many attentions for their strong light extinction,^11^ ultralightweight, and especially promising high temperature tolerance (e.g., melting point ≈4527 °C for graphite^12^). Macroscopic assemblies of carbon materials, such as aerogels, films, and blocks, can achieve the blackbody (BB)‐like spectral response with near perfect absorptance (α_solar_ > 0.95^13^) across a broadband spectrum,^11^ which are applied in photon detection,^14^ microwave absorption,^15^ and light‐to‐heat conversion.^11^, ^16^ However, the efficient spectrally selective regulation of carbon‐based materials is still extremely challenging, inducing to a high thermal emittance (ε_100_ > 0.95) and energy loss. Therefore, carbon‐based materials are commonly used as fillers or antireflection coatings (ARC) into conventional SSAs to slightly enhance the light absorptance.^17^ Up to now, the direct spectral selectivity of carbon‐based materials is still elusive.

Graphene is an atom‐thick carbon material with special 2D plane structure.[qv: 14b,18] Massive functionalities and inlayer defects of easily prepared graphene oxide (GO) dispersion provide desirable freedom for the physical or chemical regulation on graphene macroscopic assemblies with diverse optical properties.^19^ In this study, we report the reduced graphene oxide (rGO) film with excellent spectral selectivity through a simple and low‐cost sol‐gel method.^6^ The obtained rGO film possesses an appropriate and tunable lossy interference of sunlight by easily regulating the thickness and reduction level of inner 2D graphene sheets. Comparing to conventional carbon‐based black assemblies, thermal emittance of rGO film based spectrally selective absorber (rGO‐SSA: ε_100_ ≈ 0.04) is largely reduced by ≈95.8% while solar absorptance remains at a high level (α_solar_ ≈ 0.92). More importantly, rGO‐SSA exhibits high temperature tolerance at 800 °C for 96 h and a theoretical long‐term stability for 25 years at 177 °C. The broadband‐tunable cutoff wavelength of rGO‐SSA ranges from 1.1 to 3.2 µm, which is adaptive in applications of broad conditions (<100 to ≈800 °C). As a result, the efficient utilization of solar radiation of rGO‐SSA allows high surface temperature and fast surface solar steam escape speed (0.94 mg cm^−2^ s^−1^) when directly exposed to 6 sun radiation for superheated droplet boiling. This discovery will promote the development of convenient and high‐performance carbon‐based intrinsic SSAs, opening their creative applications in various solar thermal conversion areas.

## 2 Results and Discussion

### 2.1 Preparation and Characterization of rGO‐SSA

**Figure 1:**
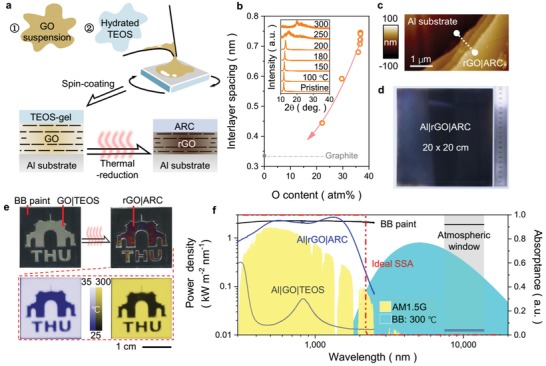
Preparation and characterization of rGO‐SSA. a) Two‐step sol‐gel spinning process of rGO‐SSA, GO film is deposited on polished Al substrate firstly and TEOS gel layer is formed on the top of Al|GO, deriving an Al|GO|TEOS sandwich. And Al|GO|TEOS is transformed into Al|rGO|ARC (i.e., rGO‐SSA) by thermal reduction (300 °C). b) Interlayer spacing of graphene oxide layer in rGO‐SSA, measured by X‐ray diffraction (inset) after different annealing temperatures. c) Atomic force microscopic (AFM) picture of rGO|ARC layers on Al. d) Large‐area rGO‐SSA(Al|rGO|ARC) with 20 × 20 cm square coverage area, produced by sol‐gel spraying method. e) Optical and IR photos of rGO‐SSA compared to a BB paint before/after thermal reduction. The upper row shows the appearance change of rGO‐SSA (patterned area) before/after thermal reduction, and surrounding area is covered with BB paint. The BB paint (Botny B‐1939) is a high‐temperature‐resistant black paint based on organic silicon. The lower row demonstrates the low thermal emittance of rGO‐SSA compared to BB paint either heating at 30 or 300 °C via IR imaging. f) Absorptance responses across UV–MIR spectrum of rGO‐SSA before/after thermal reduction are shown in gray and blue line, respectively. Power density distributions of solar radiation (AM1.5G, Air mass: 1.5 Global) and theoretical blackbody radiation (BB: 300 °C) are shown in yellow and blue block, respectively. Absorptance responses of BB paint and ideal SSA (theoretical) are shown in black line and red dashed, respectively. Gray block represents the atmospheric window across 7–14 µm for measuring thermal emittance of objects in common IR imaging.

For preparation of rGO‐SSA, GO suspension and hydrated tetraethoxysilane (TEOS) solution was successively coated on polished metallic substrate (e.g., Al), respectively, forming Al|GO|TEOS multilayer structure (**Figure** **1**a). After ≈300 °C thermal reduction for 30 min (Figure 1b), GO changes into rGO film with the interlayer spacing of inner sheets reduces from ≈0.75 to ≈0.45 nm. TEOS gel transforms to silica nanoparticles layer, which will act as ARC to reduce the reflection loss. The obtained rGO‐SSA is consisted of Al substrate, multilayer rGO film, and ARC layer (Al|rGO|ARC, Figure 1c). Height scanning of layer section shows that rGO layer is ≈100 nm in thickness and ARC layer is ≈50 nm (Figure S1, Supporting Information). Surface scanning of rGO‐SSA demonstrates the coating is uniform (Figure S2, Supporting Information), for the surface roughness is rather low (*R*_q_ ≈ 10.5 nm) and surface area difference is less than 1.5% (Figure S2, Supporting Information). In addition, large‐scale rGO‐SSA with square of 20 × 20 cm (Figure 1d) is easily obtained by this sol‐gel method. After thermal reduction, the surface color of rGO‐SSA changes from almost transparent to black (Figure 1e and Figure S3, Supporting Information), which could be resulted from strong absorption enhancement for π‐band transition in graphene lattice in the reduction process.^20^ The apparent temperature by IR camera of rGO‐SSA(Al|rGO|ARC) is much lower than conventional black aerosol coating body (BB paint) with same substrate either heating at 30 or 300 °C (Figure 1e) (Video S1, Supporting Information), indicating the selectively low infrared spectrum emittance of rGO‐SSA(Al|rGO|ARC) is highly different from BB paint and others though they both show black.

In nature, radiation wavelength distribution of sun is in 0.3–2.5 µm, which mainly ranges from ultraviolet (UV) to near‐infrared (NIR). Solar absorbers always emit thermal radiation across mid‐Infrared spectrum (MIR, 2.5–25 µm) to environment when absorbing solar radiation across UV–NIR (e.g., AM1.5G, Figure 1f), which causes inevitable thermal energy loss. For an opaque material, based on Kirchhoff's laws, the emittance of material is equal to the absorptance in a given wavelength λ, namely, ε(λ) = α(λ). Therefore, to reduce as much thermal radiation loss in MIR as possible while retaining sufficient solar radiation input in UV–NIR, absorptance α(λ) of an ideal SSA in MIR should be prohibited, forming an equivalent low emittance ε(λ). In another word, as shown with red dashed line in Figure 1f, absorptance (α(λ)) of an ideal SSA should suddenly drop from 1 to 0 right at the cutoff wavelength (λ_cut_, defined as the wavelength in which α(λ) equals to 0.5 for SSA) where power density distributions of solar radiation and absorber thermal radiation are intersected. Spectral analysis in Figure 1f shows absorptance of BB paint is nearly wavelength‐independent high (0.91–0.95) from UV to MIR, which induces the high apparent temperature detected by IR camera mentioned above (Figure 1e). On the contrary, the prepared rGO‐SSA(Al|rGO|ARC) has high absorptance across solar spectrum (≈0.92) and rather low absorptance across MIR (≈0.04), thus performing excellent spectral selectivity and low apparent temperature (Figure 1e).

### 2.2 Spectral Selectivity of Multilayer rGO Film

**Figure 2:**
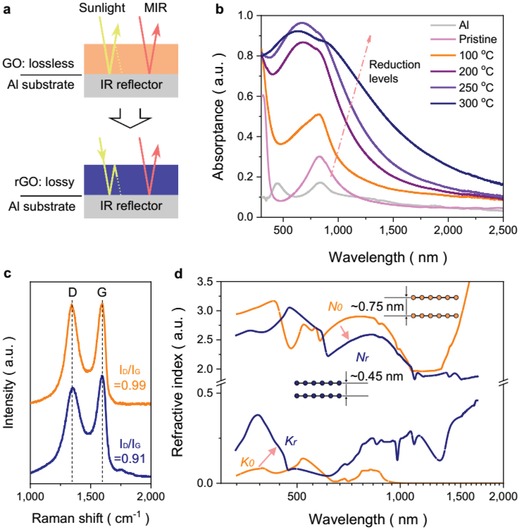
Lossy interference induced spectral selectivity of rGO film. a) Scheme of lossless interference in GO film, and lossy interference effect in rGO film. b) Solar absorptance enhancement of rGO film with different reduction temperatures. c) Raman spectra of rGO film before/after 300 °C reduction. d) Complex spectral refractive index for GO film is plotted in orange before thermal reduction (*N*_0_, *K*_0_), while in navy blue after 300 °C reduction (*N*_r_, *K*_r_).

This interesting transformation in spectral response of rGO‐SSA(Al|rGO|ARC) is probably attributed to the lossy property of rGO thin film induced by the inner structural change during thermal reduction (**Figure** **2**a). To figure out the relationship between selective solar absorptance and underlying optical property of rGO film during annealing, we have coated a GO film in thickness of ≈50 nm on Al substrate (Al|GO), and processed it at different temperatures (Figure 2b). Spectral measurement shows that bare Al substrate has solar absorptance of 0.12 and Al|GO has absorptance of 0.15, respectively. With reduction level increases, solar absorptance of Al|rGO is evidently elevating and reaches to ≈0.8 after 300 °C treatment. Initial GO sheets in film are fully coupled with oxygen functionalities, resulting a big interlayer spacing of ≈0.75 nm (Figure 1b) and high sp^3^/sp^2^ hybridization ratio (*I*_D_/*I*_G_ = 0.99, Figure 2c) of carbon atoms.^21^ After thermal reduction, most oxygen functionalities of GO are decomposed, accompanying with that sp^3^/sp^2^ hybridization ratio decreased (*I*_D_/*I*_G_ = 0.91) and interlayer spacing reduced to ≈0.45 nm. The drastically reduced interlayer spacing and decreased sp^3^/sp^2^ ratio of final rGO thin film should lead to enhancements of inter‐ and intraband transition,^20^ respectively, inducing strong light extinction in rGO film.

As complex spectral refractive indices (*N* + *iK*) of GO and rGO films are shown in Figure 2d, the real component (*N*, namely, refractive index) of rGO film is around 2.3 across measured spectrum that is similar to the value of GO film (2.5), revealing the well orientation of graphene sheets in rGO film that enables the sunlight constructive interference condition. Extinction coefficient (*K*) of rGO film is rather higher (≈0.25, averaged) than that of GO film (≈0.02, averaged), demonstrating the successful lossless (GO) to lossy (rGO) transformation with the inner structural changes. In this lossy but thin rGO film (≈100 nm), constructive sunlight interference causes strong absorption (Figure 2b) ranges across solar spectrum (UV–NIR).^22^ However, it is different to achieve interference condition for long wave light across MIR spectrum in rGO film, which is mostly reflected by Al substrate (working as IR reflector), resulting promising selective spectral response of Al|rGO (Figure 2a,b). These results have confirmed our assumption that selective absorptance of rGO‐SSA (Figure S4, Supporting Information) in this study is mainly attributed to the lossy constructive interference in rGO film induced by the inner structural change.

### 2.3 In Situ Thermal Radiation Properties

**Figure 3:**
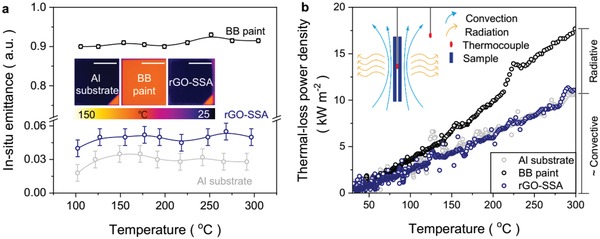
In situ characterization of thermal emittance (ε_T_) and thermal‐loss power density (*P*_loss_) under different temperatures. a) In situ ε_T_ derived by manual IR emittance modification, inset is IR photos of studied samples at heating temperature ≈100 °C. Scale bar: 1 cm. b) Calculated *P*_loss_ of studied samples from 30 to 300 °C. Inset is a scheme of testing setup, sample is gradually heated up to stable temperature ≈300 °C and for each test process, heating source is removed immediately at the beginning of temperature sampling.

The elevated‐temperature thermal emittance (ε_T_, *T* > 100 °C) of SSA is a key factor for reducing thermal radiation loss to environment, because radiation energy density increases with the fourth power of the temperature (∝*T* ^4^).^23^ Many researchers calculate elevated‐temperature thermal emittance using the absorptance data sampled at room temperature,^8^, ^10^, ^24^ which causes inevitable underestimation of emittance for higher working temperature (*T* > 100 °C). The ultralow MIR absorptance indicates the likewise low thermal (MIR) emittance of rGO‐SSA, which can be straightforwardly characterized via IR camera. Therefore, we have performed an in situ thermal emittance characterization (detected spectrum range: 7–14 µm) for absorbers (rGO‐SSA, BB paint, and Al substrate) by using reference aided IR emittance modification system (Figures S5 and S6, Supporting Information) when heating samples at working temperature (100–300 °C).^25^ Each sample has a triangular reference region (rough polyamide tape with already‐known emittance ≈0.95 adjusted by an embedded thermocouple) to modify the MIR emittance of studied region. As shown in **Figure** **3**a, rGO‐SSA retains almost the similar low thermal emittance to bare substrate (below 0.06) at different temperatures (100–300 °C), while BB paint has a thermal emittance ≈0.91 of high radiative loss property. The low thermal emittance of rGO‐SSA could mean low thermal loss to the environment, which will hide the energy on the body and enhance the solar‐thermal conversion efficiency. We have measured the areal thermal loss power density (*P*_loss_) of these three samples (rGO‐SSA, BB paint, and Al substrate) at given working temperature by a free cooling test at a beginning temperature of 300 °C in atmosphere (inset in Figure 3b), which evaluates the ability of reducing thermal radiation loss to environment. *P*_loss_ is approximated by the free cooling power density (*P*_cool_)^26^

(1) $$P_{\text{loss}}\approx P_{\text{cool}}=cm\Delta T\text{/}S\Delta t$$

where *c* is specific heat capacity, *m* is mass, *S* is the surface area, Δ*t* is the cooling time difference, and Δ*T* is the real‐time temperature difference of absorber.

Through the real‐time temperature records of sample and environment (Figure S7, Supporting Information), calculated *P*_loss_ of BB paint is higher than that of Al substrate and rGO‐SSA from room temperature to 300 °C that indicates the less thermal loss of rGO‐SSA for the low thermal emittance (Figure 3b). Specifically, *P*_loss_ of rGO‐SSA at 300 °C is about 11.1 kW m^−2^, which saves ≈37% of thermal energy comparing to BB paint (17.5 kW m^−2^). These results indicate that thermal radiation loss to the environment is effectively reduced and solar‐thermal conversion efficiency is indeed enhanced by rGO‐SSA even at high working temperatures.

### 2.4 Broadband‐Tunable Spectral Selectivity toward Multipurpose Applications

**Figure 4:**
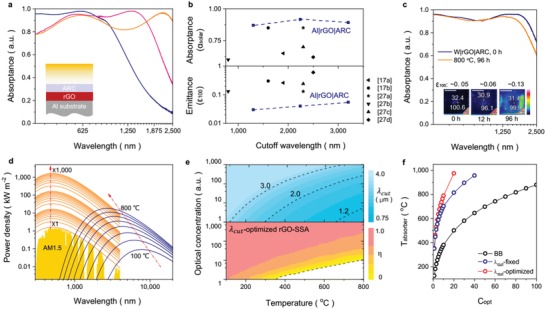
Spectrally selective response of rGO‐SSAs. a) Spectral absorptance of rGO‐SSA(Al|rGO|ARC) with rGO film of different thicknesses (rGO ≈ 50 nm for blue line, rGO ≈ 100 nm for pink line, and rGO ≈ 150 nm for orange line). b) Comparison of total solar absorptance (α_solar_) and thermal emittance (ε_100_) of rGO‐SSA(Al|rGO|ARC) to previously reported carbon‐containing SSAs: CuCoO+GO,[qv: 17a] pigment particles+graphene,[qv: 17b] electrophoretically deposited CNTs,[qv: 25a] amorphous carbon|Au,[qv: 25b] NiO+CNTs,[qv: 25c] aligned CNTs.[qv: 25d] c) Spectral absorptance and IR photos (inset) of rGO‐SSA(W|rGO|ARC) before/after annealing at 800 °C for 96 h. Scale bar: 1 cm. d) Overlap between solar radiation (*C*_opt_, 1–1000) and thermal radiation (*T*_w_, 100–800 °C) across UV–MIR. e) Cutoff wavelength optimization of rGO‐SSA and corresponding solar thermal efficiency under isolated condition in vacuum. f) Theoretical balanced temperatures in vacuum isolated condition. BB, rGO‐SSA_λcut‐fixed_, and rGO‐SSA_λcut‐optimized_ are shown in black, blue, and red lines, respectively.

Meanwhile, in solar‐thermal conversion process, the increased surface temperature implies the thermal radiation of SSAs blueshifted to higher energy spectrum (i.e., shorter wavelength). Thus, radiative power density distributions of sunlight and absorber are intersected at different wavelengths depending on working temperature, requiring variable λ_cut_ for designing the spectral response of absorber. With the simple solution methods in preparation, λ_cut_ of rGO‐SSA can be easily controlled by mainly regulating the constructive interference with thicknesses change of rGO film. As shown in **Figure** **4**a, λ_cut_ of rGO‐SSAs(Al|rGO|ARC) ranging from 1.3 to 3.2 µm are fabricated with the thickness of rGO films changed from ≈50 to ≈150 nm (Figure 1c, Figures S1b and S8, Supporting Information). For λ_cut_ varying from 1 to 3 µm, all rGO‐SSAs(Al|rGO|ARC) sustain the lowest thermal emittance (ε_100_ < 0.05), and still approach the highest solar absorptance (α_solar_ ≈ 0.92) (Figure 4b and Figure S9, Supporting Information), which is outstanding to the performances of reported carbon‐containing SSAs[qv: 17a,b,27] as far as we know, and is comparable to the best achieved of other SSAs by sol‐gel method (Table S1, Supporting Information).^28^ To strengthen the high‐temperature stability of rGO‐SSA, we have used tungsten to replace Al as IR reflector (Figure 4c). A customized accelerating aging test is also performed at 800 °C under argon protection for as‐prepared rGO‐SSA(W|rGO|ARC), the spectral characterizations before/after aging test (Figure 4c, Figure S10 and Table S2, Supporting Information) show that the performance criterion (PC) value for rGO‐SSA(W|rGO|ARC) is 0.008 after 12 h annealing and is 0.041 for 96 h annealing. Note that PC ≤0.05 is the qualified scale, so rGO‐SSA(W|rGO|ARC) can be practically used for 96 h (at least) at 800 °C (details in Section S2, Supporting Information). This is a considerable high level, for the thermal stability performance of most of sol‐gel SSAs covering always the low‐temperature (*T <* 100 °C) scale (Table S1, Supporting Information). The theoretical long‐term stability of rGO‐SSA(W|rGO|ARC) is calculated in the basis of the high‐temperature performance, and shows that rGO‐SSA(W|rGO|ARC) could be practically used for ≈25.5 years at 177 °C (Section S2, Supporting Information).

rGO‐SSA allows excellent high‐temperature stability in combination of broadband‐tunable selectivity, making it adaptive in multipurpose solar harvesting systems. In practical systems (Figure 4d), solar absorbers are usually used in various optical concentration indices (*C*_opt_, 1–1000) and working temperatures (*T*_w_, 100–800 °C). When absorber is thermally isolated in vacuum, the solar thermal conversion efficiency (η) can be estimated by (details in Section S3, Supporting Information)^29^

(2) $$\eta =\frac{\alpha_{\text{solar}}\cdot C_{\text{opt}}\cdot E_{\text{AM1}.\text{5G}}-\varepsilon_{T_{\text{w}}}\cdot \sigma \left(T_{\text{w}}^{4}-T_{\text{ambient}}^{4}\right)}{C_{\text{opt}}\cdot E_{\text{AM1}.\text{5G}}}=\alpha_{\text{solar}}-\varepsilon_{T_{\text{w}}}\cdot \frac{T_{\text{w}}^{4}-T_{\text{ambient}}^{4}}{C_{\text{opt}}\cdot \beta \cdot T_{\text{s}}^{4}}$$

where *T*_ambient_ is the ambient temperature, *T*_s_ = 5770 K is the solar temperature, σ = 5.6696 × 10^−8^ W m^−2^ K^−4^ is the Stefan–Boltzmann constant, and β = 2 × 10^−5^ is a scaling factor for fitting AM1.5G power spectrum (*E*_AM1.5G_).

Note that solar absorptance (α_solar_) controls the input solar flux, and thermal emittance (*ε_T_*_w_) determines the thermal radiation loss when absorber reaches to the theoretical balanced temperature under certain concentrated sunlight (*C*_opt_). Thus, the optimized balanced temperature of SSA should reach to the working temperature (*T*_w_) where η = 0 under concentrated sunlight (*C*_opt_), which will maximize the utilization of solar energy.

We should maneuver the trade‐off between solar input and radiative loss considering the actual working condition (*C*_opt_, *T*_w_) for maximizing the solar thermal conversion (Figure 4d). Selecting shorter λ_cut_ not only means less input solar flux (α_solar_ decreases), but also less thermal radiation loss at high temperature (*ε_T_*_w_ decreases). For low *T*_w_ systems (e.g., solar‐thermal deicing,[qv: 1b] water heating^6^), thermal radiation is negligible compared to solar input, thus longer λ_cut_ is desirable for maximizing the solar absorptance. However, in high *T*_w_ systems (e.g., solar thermophotovoltaic) with thermal radiation seriously increased, the appropriate λ_cut_ should be shorter for reducing thermal radiation loss. Theoretically, in a given working condition (with specific *C*_opt_, and *T*_w_), the cutoff wavelength λ_cut_(*C*_opt_, *T*_w_) (Figure 4e) can be explicitly defined as (details in Section S3, Supporting Information)^2^

(3) $$\lambda_{\text{cut}}\left(C_{\text{opt}},T_{\text{w}}\right)=\frac{c_{2}\cdot \left(T_{\text{s}}-T_{\text{w}}\right)}{T_{\text{s}}\cdot T_{\text{w}}\cdot \ln\left(\frac{1}{\beta \cdot C_{\text{opt}}}-1\right)}$$

To investigate the applicability of rGO‐SSA for different solar concentration energy systems, we simulated three absorbers (BB, rGO‐SSA*_λ_*_cut‐fixed_, and rGO‐SSA*_λ_*_cut‐optimized_) in vacuum isolated condition. For BB absorber, α(λ) = ε(λ) = 1.0, which is wavelength independent. rGO‐SSA*_λ_*_cut‐fixed_ has a fixed λ_cut_ of 2.2 µm, so the absorptance and emittance are expressed as step‐function

(4) $$\alpha \;\left(\lambda \right)=\;\varepsilon \;\left(\lambda \right)=\{\begin{array}{c} 0.9,\;\lambda <2.2\\ 0.05,\;\lambda \geq 2.2 \end{array}$$

rGO‐SSA*_λ_*_cut‐optimized_ has an optimized λ_cut_ = λ_cut_(*C*_opt_, *T*_w_), then the absorptance (emittance) is

(5) $$\alpha \;\left(\lambda \right)=\;\varepsilon \;\left(\lambda \right)=\{\begin{array}{c} 0.9,\;\lambda <\lambda_{\text{cut}}\left(C_{\text{opt}},T_{\text{w}}\right)\\ 0.05,\;\lambda \geq \lambda_{\text{cut}}\left(C_{\text{opt}},T_{\text{w}}\right) \end{array}$$

When absorber only stores solar thermal energy but not converts it out of system, the theoretical balanced temperature of absorber under concentrated sunlight is correspondent to the working temperature where the solar thermal conversion efficiency η = 0, which is plotted in black dot line for rGO‐SSA*_λ_*_cut‐fixed_ (Figure 4e). Specially, we have compared the solar thermal efficiency of these absorbers (BB, rGO‐SSA*_λ_*_cut‐fixed_, and rGO‐SSA*_λ_*_cut‐optimized_) under concentrated sunlight (Figure 4f). The result in Figure 4f shows that the balanced temperatures of rGO‐SSA*_λ_*_cut‐fixed_ and rGO‐SSA*_λ_*_cut‐optimized_ are almost two times higher than that of BB absorber. For rGO‐SSA*_λ_*_cut‐fixed_, growing speed of balanced temperature becomes slower than rGO‐SSA*_λ_*_cut‐optimized_ when *C*_opt_ > 10. Both of rGO‐SSA*_λ_*_cut‐optimized_ and rGO‐SSA*_λ_*_cut‐fixed_ approach ≈800 °C under much lower *C*_opt_ than BB absorber (×75, ×18, and ×10 for BB, rGO‐SSA1, and rGO‐SSA2, respectively). These analyses indicate selecting λ_cut_(*C*_opt_, *T*_w_) of rGO‐SSA according to optical concentration and working temperature will approach the best performance in solar energy harvesting systems. Above all, as‐prepared rGO‐SSAs already meet these requirements for the broadband‐tunable spectral selectivity (λ_cut_ 1.3–3.2 µm, with α_solar_ ≈ 0.92 and ε_100_ ≈ 0.04) and high temperature stability (800 °C).

### 2.5 Ultrafast Steam Escape Using rGO‐SSA Surface

**Figure 5:**
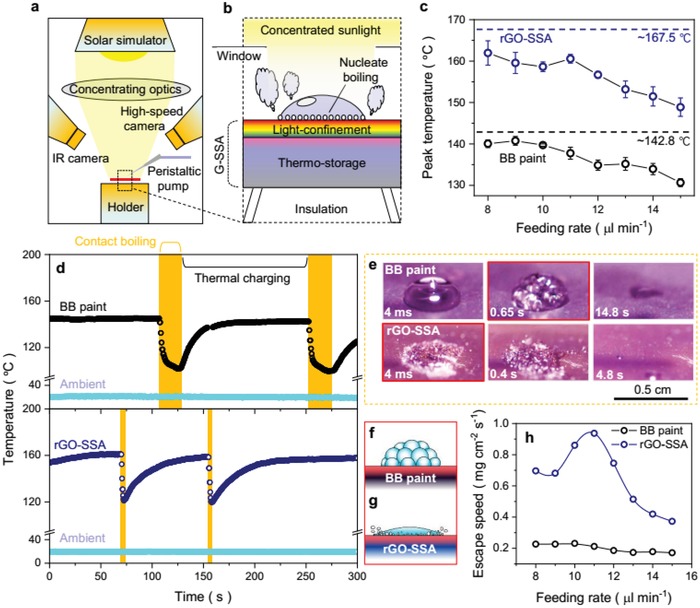
Ultrafast steam escape on rGO‐SSA surface under concentrated solar radiation. a) Testing setup of solar steam escape: rGO‐SSA is placed on a thermal insulating holder and exposed under concentrated sunlight, and a peristaltic pump delivering intermittent droplets onto rGO‐SSA surface while optical and thermal information during droplet boiling is recorded by high‐speed camera and IR camera, respectively. b) Scheme of steam escape by ultrafast nucleated contact boiling on rGO‐SSA surface. rGO|ARC in rGO‐SSA functions as an ultrathin (≈150 nm) light‐confinement layer which efficiently absorbs sunlight and transfers thermal energy, while bulk Al substrate plays the part of thermo‐storage which stores thermal energy as lattice vibration and electron intraband transition. When water droplet drops onto rGO‐SSA surface, heat power in Al rapidly transfers into water droplet through massive nucleate sites (provided by porous ARC layer) and short conduction distance (rGO|ARC). c) Peak temperature of absorber surface (blue dot is for rGO‐SSA and black dot is for BB paint) in feeding rates from 8 to 15 µL min^−1^. Dashed line is the balanced temperature of the absorber in 6 sun without droplet feeding. d) Real‐time surface and ambient temperatures of the absorber in feeding rate of 8 µL min^−1^. Orange block depicts the contact boiling period and the temperature growing period after droplet dried out is named as thermal charging period. e) Optical photos of contact boiling period on absorber surface, starting point of boiling is 650 ms or BB paint, and is 4 ms for rGO‐SSA. f) Scheme of film boiling on BB paint. g) Scheme of nucleate boiling on rGO‐SSA. h) Escape speed for a single droplet on rGO‐SSA surface (blue point line) and BB paint surface (black point line).

Generating high temperature and big flux steam is significant in many solar‐thermal applications, including steam sterilization,^30^ steam turbine generators,^31^ and so on. The low thermal emittance rGO‐SSA can produce high substrate temperature under solar irradiation (**Figure** **5**a,b). Under concentrated solar irradiance (*C*_opt_ ≈ 6), rGO‐SSA reaches a balanced temperature of ≈167.5 °C (Figure 5c), which is 24.7 °C higher than BB paint without spectral selectivity (≈142.8 °C). When the water droplet reaches the surface of rGO‐SSA, the drastic boiling occurs soon. With thermal energy in Al rapidly conducting to nucleate surface through ultrathin rGO|ARC layer (≈150 nm), continuous boiling will last until the droplet dried out. However, droplet boiling induced steam escape on BB paint surface is seriously hindered for the low solar thermal conversion and long‐distance (≈20 µm) heat transfer nature. When feeding droplet with 8 µL min^−1^ (Figure 5d), surface temperature of BB paint drops by ≈47 °C in 14.8 ± 1.2 s, while rGO‐SSA suddenly drops by ≈43 °C in 4.8 ± 0.7 s, demonstrating rGO‐SSA transforms water droplet into saturated steam much faster than BB paint (≈3 times).

Across contact boiling period, the droplet on rGO‐SSA reaches visible boiling in 4 ms (Figure 5e), which is ≈162 times faster than BB paint (650 ms). From bubbling shape, we can see BB paint enters film boiling state prematurely (Figure 5f), confirming the worse heat flux capacity of BB paint surface. In contrast, rGO‐SSA exhibits continuous nucleate boiling state (Figure 5g), which provides sufficient heat flux while droplet boiling.^32^ Based on the recorded dried out time (Figure S11, Supporting Information) and droplet mass (13.4 ± 0.5 mg), we have calculated the steam escape speed for the two samples (Figure 5h). When feeding rate is 11 µL min^−1^, ultrafast steam escape (0.94 mg cm^−2^ s^−1^) on rGO‐SSA surface is achieved, which is ≈4 times over the maximum escape speed (0.23 mg cm^−2^ s^−1^) of BB paint surface. All these results show a promising way of rGO‐SSA for generating big flux and high temperature steam, in which we have realized ultrafast solar steam escape by making use of the spectral selectivity and ultrathin natures of rGO‐SSA.

## 3 Conclusion

In summary, we have observed that hundred nanometers thin film rGO on Al substrate can achieve significant lossy interference of sunlight and developed a high‐performance rGO‐SSA through a simple sol‐gel method. rGO‐SSA not only enables low cost and easily scalable production, but also exhibits excellent spectral selectivity (α_solar_ ≈ 0.92 and ε_100_ ≈ 0.04), high temperature tolerance (≈800 °C), and broadband tunability (λ_cut_, 1.3–3.2 µm), indicating rGO‐SSA is desirable for various solar energy systems. Making use of the superior solar thermal conversion and ultrathin heat transporting abilities of rGO‐SSA, an ultrafast escaping steam flux (0.94 mg cm^−2^ s^−1^) directly under 6 sun is realized. These findings of strong light extinction of graphene film will promote versatile designs of graphene‐based nanostructures for light‐harvesting, optoelectronic, and thermal‐photonic applications.

## 4 Experimental Section

##### Fabrication of rGO‐SSA

GO suspension (15 mg g^−1^) was prepared by modified Hummers method.^16^ The resulting suspension was centrifuged at 10 000 rpm to remove multilayer GO flakes. Solution of hydrated TEOS was synthesized by sol‐gel process (TEOS:ethanol:H_2_O:HCL = 15:40:5:0.07, volume ratio). Diluted GO (1:10, *V*_GO_:*V*_ethanol_) and hydrated TEOS were coated onto polished substrates (aluminum, or tungsten) by spin‐coating (4000 rpm). As‐prepared tandem absorber (substrate|GO|TEOS) was annealed at 300 °C on a hot plate to get rGO‐SSA (substrate|rGO|ARC). Large‐scale rGO‐SSA was fabricated by sol‐gel spraying process, diluted GO (1:50, *V*_GO_:*V*_ethanol_) and TEOS solution (1:50, *V*_TEOS_:*V*_ethanol_) were sprayed onto polished aluminum substrate, respectively, by an air brush (UA‐601G, U‐STAR), and then annealed at 300 °C forming large‐area rGO‐SSA.

##### Optical Characterization

Spectral reflectance of rGO‐SSA was measured in UV–NIR (0.3–2.5 µm, measured in normal direction) using Universal‐Measurement‐Spectrophotometer (Cary 7000, AGILENT) equipped with an integrating sphere. A silica protected aluminum reflector (ME1‐G01, THORLABS) was used as a reference. Raman spectra were measured with a HORIBA Raman spectrometer (LabRAM HR Evolution). Refractive index measurement was taken for rGO film using spectroscopic ellipsometer (0.6–2 µm, Horiba UVISEL Plus) based on point‐by‐point *NK* fitting method.

##### Structural Characterization

Surface and layer section morphology was measured by atomic force microscopy (Innova AFM, BRUKER). X‐ray diffraction (XRD) measurement was taken using a BRUKER D2 diffractometer with Cu Kα radiation (λ = 1.5406 Å). Atomic fraction of oxygen was measured by energy‐dispersive spectroscopy (EDS, JSM‐7500F).

##### Thermal Stability Measurement

rGO‐SSA was first sealed in a 1 in. diameter quartz tube with argon protection, and then gradually heated to the desired temperature (≈5 °C min^−1^, 800 °C) using tubular furnace (OTF‐1200X, HF‐Kejing) and naturally cooling with furnace after 96 h annealing.

##### Solar Steam Escape Measurement

Testing was performed on a solar simulator (CEL‐HXF300, CEAULIGHT) with an optical filter for the standard AM1.5 G spectrum. A Fresnel lens (diameter ≈ 60 mm, focal distance ≈ 80 mm) was used to concentrate sunlight. IR camera (Tix640, FLUKE) and high‐speed camera (V611, PHANTOM) were employed to record droplet vaporization process. Feeding rate of droplet was controlled by a peristaltic pump (BT101, LEAD FLUID). Droplet mass and actual feeding rate were adjusted by an accurate electronic balance (ME104E, METTLER TOLEDO).

## Conflict of Interest

The authors declare no conflict of interest.

## Supporting information

Supporting Information

Supplemental Video 1
