# Supplementary material for: Reduced Graphene Oxide–Based Spectrally Selective Absorber with an Extremely Low Thermal Emittance and High Solar Absorptance
Source: Adv Sci (Weinh). 2020 Feb 27;7(8):1903125. doi: 10.1002/advs.201903125 (PMC7175286; doi:10.1002/advs.201903125)
Supplement: Supplementary file 1 — Supporting Information [file ADVS-7-1903125-s001.pdf]

Copyright WILEY-VCH Verlag GmbH & Co. KGaA, 69469 Weinheim, Germany, 2020.

ADVANCED SCIENCE
Open Access

Supporting Information

for *Adv. Sci.,* DOI: 10.1002/advs.201903125

Reduced Graphene Oxide–Based Spectrally Selective Absorber with an Extremely Low Thermal Emittance and High Solar Absorptance

Qihua Liao, Panpan Zhang, Houze Yao, Huhu Cheng, Chun Li, and Liangti Qu**

Copyright WILEY-VCH Verlag GmbH & Co. KGaA, 69469 Weinheim, Germany, 2020.

Supporting Information

Reduced Graphene Oxide based Spectrally Selective Absorber with an Extremely Low Thermal Emittance and High Solar Absorptance

Qihua Liao[1], Panpan Zhang[1], Houze Yao[1], Huhu Cheng[1,], Chun Li[2] and Liangti Qu[1,2,3*]*

Section 1. Total Solar Absorptance (α_{solar}) and Thermal Emittance (ε_T)

For an opaque material, wavelength dependent absorptance $\alpha(\lambda)=1-\rho(\lambda)$, $\rho(\lambda)$ is spectral reflectance. Then total solar absorptance can be expressed as:[1]

$$\alpha_{solar} = \frac{\int d\lambda \cdot \alpha(\lambda) \cdot E_{Am1.5G}(\lambda)}{\bar{E}_{Am1.5G}} \tag{S1}$$

Where $E_{Am1.5G}(\lambda)$ is the power density distribution of solar radiation (AM1.5G, Air mass:1.5 Global spectrum), $\bar{E}_{Am1.5G}=1.00\ kW\ m^{-2}$, which is the total solar power density.

Using Kirchhoff's laws, emittance is equal to the absorptance in a given wavelength, namely $\varepsilon(\lambda)=\alpha(\lambda)$. For an absorber at working temperature T, its thermal radiation emittance can be expressed as[1]:

$$\varepsilon_T = \frac{\int d\lambda \cdot \varepsilon(\lambda) \cdot E_{BB}(\lambda,T)}{\bar{E}_{BB\langle T\rangle}} \tag{S2}$$

Where $E_{BB}(\lambda, T)$ is power density distribution of BB surface:[2]

$$E_{BB}(\lambda,T)= c_1\lambda^{-5}\left[e^{\left(\frac{c_2}{\lambda T}\right)}-1\right]^{-1} \tag{S3}$$

In which $c_1=3.7405 \times 10^8\ W\ \mu m^4\ m^{-2}$ and $c_2=1.43879 \times 10^4\ \mu m\ K$, which are Planck's first and second radiation constants, respectively. And $\bar{E}_{BB<T>}$ is total thermal radiation power density:

$$\bar{E}_{BB\langle T\rangle} = \sigma T^4 \tag{S4}$$

Where $\sigma=5.6696 \times 10^{-8}\ W\ m^{-2}\ K^{-4}$ is the Stefan-Boltzmann constant.

Section 2. Long-term stability and degradation mechanism of rGO-SSA

Towards the long-term stability evaluation of low-temperature SSAs for domestic solar water heating systems, the accelerating aging method has been introduced at 1994 in the work of International Energy Agency (IEA) Task X.[3] Considering the 25 years stable performance (degradation of solar fraction less than 5 %) of absorber under working condition of water heating, this accelerating aging method introduces a performance criterion (PC) function for evaluate the suitability of SSAs for long-term domestic solar water heating application:

$$PC = -\Delta\alpha_{solar} + 0.25\Delta\varepsilon_{100} \leq 0.05 \quad \text{(S5)}$$

Where $\Delta\alpha_{solar}=\alpha_{solar}(aged)-\ \alpha_{solar}(unaged)$ is the change of solar absorptance, and $\Delta\varepsilon_{100}=\varepsilon_{100}\ (aged)-\ \varepsilon_{100}\ (unaged)$. This criterion function is widely-used for standard evaluation of SSAs for commercial low-temperature water heating application, however, the weight of $\Delta\varepsilon_{100}$ should be modified as 0.5 for mid-temperature ($100\ ^oC<T<400\ ^oC$) to high-temperature ($T>400\ ^oC$) applications: [4]

$$PC = -\Delta\alpha_{solar} + 0.5\Delta\varepsilon_{100} \leq 0.05 \quad \text{(S6)}$$

In this work, we use the customized accelerating aging method which is widely-used in literature[4] to evaluate the high-temperature stability of rGO-SSA, in which we select a proper annealing temperature and time under argon protection to quickly approach the failure limitation ($PC=0.05$), then measure the absorptance and emittance before/after annealing test and calculate the PC function. We have tested the high temperature stability of rGO-SSA by annealing two samples (W|rGO|ARC1 and W|rGO|ARC2) simultaneously at 800 ^oC under argon protection for 12 and 96 hours respectively. As summarized in Table S2, the PC function is 0.008<<0.05 for 800 ^oC aging for 12 hours (W|rGO|ARC1), which means the rGO-SSA(W|rGO|ARC1) is well qualified for working temperature 800 ^oC and lifetime 12 hours. And the PC function is 0.041<0.05 for 800 ^oC aging for 96 hours (namely 4 days),

indicating the W|rGO|ARC2 is also qualified but the working condition of 800 °C for 96 hours under argon protection is very closed the failure limitation of rGO-SSA(W|rGO|ARC2).

Certainly rGO-SSA can be used for much more long-term lifetime when decreasing the working temperature, the lifetime can be extended to even 25 years (~9125 days) under sufficient working temperature:

The accelerating aging is designed based on the Arrhenius' relationship,[5] in which the aging degradation mechanism is simplified as a temperature dependent thermal diffusion process. Assuming the thermal diffusion coefficient of absorber is constant over different temperatures, then absorber annealing at higher temperature will degrade faster than annealing at lower temperature, the acceleration of failure time under high temperature is defined as:[5]

$$a_n = \frac{t_{ref}}{t_n} = exp\left[\frac{E_T}{R}\left(\frac{1}{T_{ref}} - \frac{1}{T_n}\right)\right] \quad \text{(S7)}$$

Where t_{ref} is the time for approaching the failure criterion under low temperature T_{ref}, t_n is the time for approaching the failure criterion under high temperature T_n, E_T is the activation energy according to Arrhenius and R is the ideal gas constant ($R=8.314\ J\ K^{-1}\ mol^{-1}$). Therefore, for a verified high temperature result (T_n, t_n), the long-term service time under lower temperature (likewise t_{ref}) can be estimated by:

$$t_{ref} = t_n exp\left[\frac{E_T}{R}\left(\frac{1}{T_{ref}} - \frac{1}{T_n}\right)\right] \quad \text{(S8)}$$

Therefore, using this acceleration relationship, we can predict the failure time t_n of rGO-SSA under low temperature T_{ref} =177 °C given a verified higher temperature aging result (800 °C, 4 d). According to the recommend criterion in the literature,[5] the activation energy of rGO-SSA under argon protection is estimated to be ~50 kJ mol^{-1}, thus, for working condition of 177 °C under argon protection, the service time is predicted as ~9375 days (25.5 years).

We further analyze the mechanism for degradation of rGO-SSA at 800 °C. Generally, the degradation of SSA at high temperature could be attributed to spontaneous thermal diffusion

of molecules and atoms, surface oxidation or low coating adhesion-induced ingredients change and so on.[4] First, in this study, For the melting temperature of SiO2 (~1723 ^{o}C), C (~4527 ^{o}C) and W (~3422 ^{o}C) are considerably higher than the annealing temperature (800 ^{o}C), high temperature thermal diffusion of molecules and atoms could be negligible. Secondly, the annealing test is carried under argon protection, and the failure sample sustains the smooth planar surface which indicating the surface oxidation is not evident, so the degradation from oxidation could be small either.[6] Thus, the potential mechanism of degradation of rGO-SSA could be the relatively low coating adhesion, which could be induced by the impurities and voids in the interface[7] and the intrinsically low interfacial affinity of rGO with tungsten.

In conclusion, the high temperature stability of rGO-SSA in this early stage is at a considerable value and theoretical long-term stability at 177 ^{o}C for 25.5 years, indicating the long-term stability of rGO-SSA.

Section 3. Solar-thermal Conversion Efficiency Optimization

When absorbing solar flux, absorbers losing thermal energy to environment simultaneously (for conduction, convection and radiation), only residual part of solar thermal energy is stored in absorbers. SSAs are mostly used in vacuum systems for eliminating thermal losses from conduction and convection. Assuming an isolated SSA in vacuum (conduction and convection are negligible) under concentrated sunlight (with $E_{AM1.5G}$ spectrum and concentration index C_{opt}), solar thermal efficiency (η) can be expressed as:

$$\eta = (\bar{E}_{absorb} - \bar{E}_{loss}) / \bar{E}_{input} \tag{S9}$$

In which, for our preset condition, overall input energy of system $\bar{E}_{input}$ can be estimated by:

$$\bar{E}_{input} = \int C_{opt} \cdot E_{Am1.5G}(\lambda) \cdot d\lambda = C_{opt} \cdot \bar{E}_{Am1.5G} \tag{S10}$$

And absorb-in energy of system $\bar{E}_{absorb}$ merely dependents on the absorptance of SSA to the input spectrum:

$$\bar{E}_{absorb} = \int \alpha(\lambda) \cdot C_{opt} \cdot E_{Am1.5G}(\lambda) \cdot d\lambda = \alpha_{solar} \cdot C_{opt} \cdot \bar{E}_{Am1.5G} \tag{S11}$$

And energy loss of system $\bar{E}_{loss}$, in this case, is only caused by thermal emission:

$$\bar{E}_{loss} = \int \varepsilon(\lambda) \cdot [E_{BB}(\lambda, T_w) - E_{BB}(\lambda, T_{ambient})] \cdot d\lambda = \varepsilon_{T_w} \cdot \sigma(T_w^4 - T_{ambient}^4) \tag{S12}$$

Where ε_{Tw} is the emittance of SSA at working temperature T_w, and the ambient temperature is $T_{ambient}$.

From Supporting Equations (S9) ~ (S12), we conclude that, in vacuum isolated systems, solar thermal efficiency η is (C_{opt}, T_w) dependent:

$$\eta = \alpha_{solar} - \varepsilon_{T_w} \cdot \frac{\sigma(T_w^4 - T_{ambient}^4)}{C_{opt} \cdot \bar{E}_{Am1.5G}} \tag{S13}$$

In which it is worthy to note that α_{solar} and ε_{Tw} are strongly correlated to the wavelength dependent absorptance of SSA, $\alpha(\lambda)$, seeing Supporting Equations (S1) and (S2). For an ideal SSA with specific cutoff wavelength λ_{cut}, we describe its $\alpha(\lambda)$ as a step function:[8]

$$\alpha(\lambda) = \begin{cases} 1, & \lambda < \lambda_{cut} \\ 0, & \lambda \geq \lambda_{cut} \end{cases} \tag{S14}$$

As we mentioned earlier, increasing working temperature induces blue-shifting $E_{BB}(\lambda, T_w)$, so $E_{BB}(\lambda, T_w)$ will intersect with solar spectrum $E_{AM1.5G}(\lambda)$ in different wavelengths. Hence, λ_{cut} of SSA should be chosen as this intersection wavelength. However, as shown in Figure 4d, $E_{AM1.5G}(\lambda)$ is a discrete power distribution for atmospheric absorption band (basically from H_2O, O_3 and CO_2[9]) from the earth, so getting an analytic solution of intersection wavelength is impossible unless we approximate $E_{AM1.5G}(\lambda)$ with blackbody distribution.

The sun is a blackbody radiation source with surface temperature (T_s) of ~5770 K, power density distribution on solar surface is:

$$E_{BB}(\lambda, 5770K) = c_1\lambda^{-5}\left[e^{\left(\frac{c_2}{\lambda\cdot 5770}\right)} - 1\right]^{-1} \quad \text{(S15)}$$

When arrives at the earth, solar power is largely decreased but the relative intensity remains the same. So we can simply use a scaling factor to match $E_{BB}(\lambda, 5770K)$ to AM1.5G solar spectrum $E_{AM1.5G}(\lambda)$:

$$E_{Am1.5G}(\lambda) \approx \beta \cdot E_{BB}(\lambda, 5770K) \quad \text{(S16)}$$

In which $\beta = 2 \times 10^{-5}$ is a scaling factor, and it turns out they match well with each other (Fig. 4d). Finally, based on Supporting Equations (S3), (S10), (S15) and (S16), we get analytical solution of λ_{cut} as the intersection wavelength of $C_{opt} \times \beta \times E_{BB}(\lambda, 5770K)$ and $E_{BB}(\lambda, T_w)$:

$$\lambda_{cut}(C_{opt}, T_w) = \lambda_{\substack{C_{opt}\cdot\beta\cdot E_{BB}(\lambda,T_s) \\ = E_{BB}(\lambda,T_w)}} = \frac{c_2\cdot(T_S - T_W)}{T_S\cdot T_W\cdot \ln\left(\frac{1}{\beta\cdot C_{opt}} - 1\right)} \quad \text{(S17)}$$

Note that λ_{cut} is fixed as 4 μm when its calculated value exceeds 4 because $E_{AM1.5G}(\lambda)$ almost entirely distributed across 0.3~4 μm after atmospheric absorption.[9] And using Supporting Equations (S4) and (S13), the corresponding solar thermal efficiency can be further simplified as:

$$\eta = \alpha_{solar} - \varepsilon_{T_w} \cdot \frac{T_W^4 - T_{ambient}^4}{C_{opt}\cdot\beta\cdot T_S^4} \quad \text{(S18)}$$

In the basis of Equations above all, we have performed a numerical analysis on Matlab R2017b software, and the results suggest that 1~3 μm is the practical λ_{cut}-scale for low-to-high temperature systems.

Figures and Tables

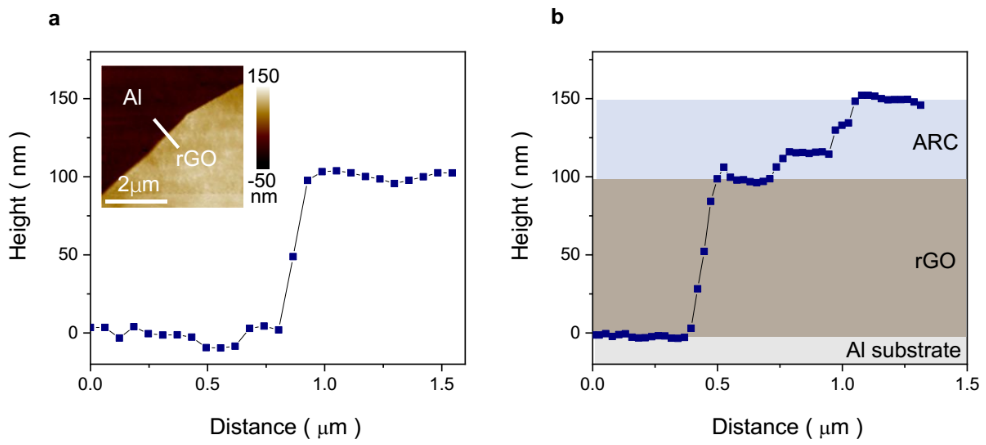

Figure S1. Layer thickness characterization of rGO-SSA(Al|rGO|ARC). (a) The height scanning of the layer section of Al|rGO; **(b)** The height scanning of the layer section of Al|rGO|ARC. It shows the first layer of rGO is ~100 nm thick and the second layer of ARC is ~50 nm thick. The polished Al substrate is a 20 x 20 x 1 mm square sheet with reflectivity over 96 %.

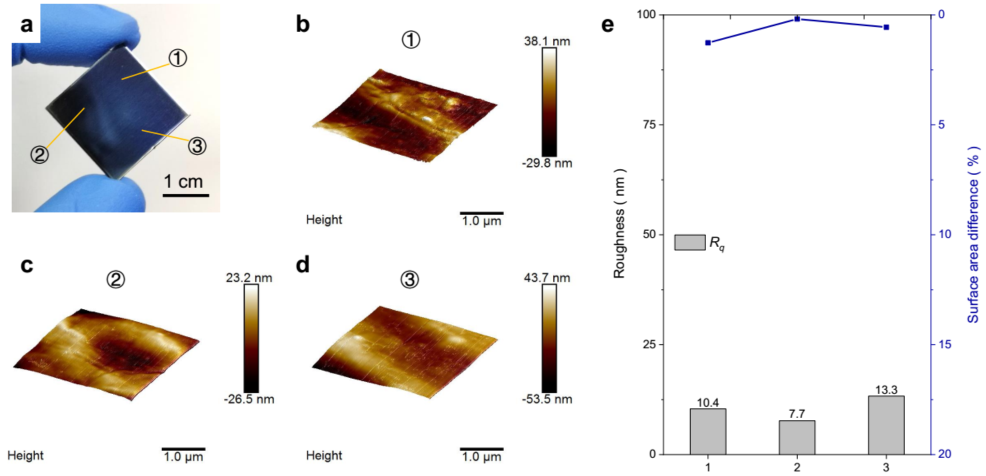

Figure S2. Characterization of the uniformity of rGO-SSA. (a) A digital photo of the as-prepared rGO-SSA under indoor illumination. **(b)**, **(c)**, **(d)** are the three-dimensional morphology figures of the randomly selected first, second, and third spot respectively, the scanning area of each spot is a 5x5 μm square. **(e)** the comparison of surface roughness R_q and surface area difference of the selected three spots.

We have measured the microscale morphology of the rGO-SSA via AFM on different regions of samples and evaluated their surface roughness over an 5x5 μm area (Figure S2). As summarized in Figure S2e, the microscale morphology of rGO-SSA is smooth and the root mean square roughness R_q of region 1, 2, 3 is about 10.4, 7.7 and 13.3 nm, respectively. Meanwhile, the surface differences of these regions are no more than 1.5 % (1.3 %, 0.2 % and 0.6 % respectively), demonstrating the planar nature of rGO-SSA surface. In conclusion, our characterization shows that rGO-SSA has smooth surface and uniform microscale morphology.

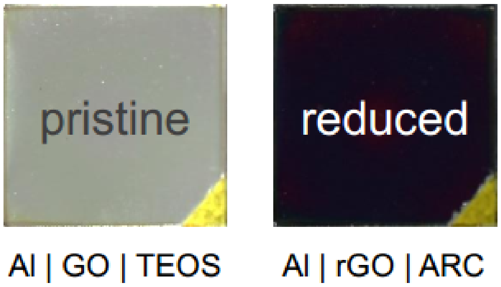

Figure S3. Optical pictures of rGO-SSA before/after thermal reduction (300 °C). Sample size: 2 x 2 cm square. Before thermal reduction, pristine Al|GO|TEOS sandwich weakly absorbs visible light (380 ~780 nm), so the optical appearance is closed to bare Al substrate (reflective, with metallic lustre). After thermal reduction, GO layer is reduced into rGO layer and TEOS is sintered into ARC layer, strong light extinction, appropriate thickness (~100 nm) of rGO layer and anti-reflection modification of ARC jointly result in the black appearance of rGO-SSA(Al|rGO|ARC) which exhibits over 0.9 absorptance across visible spectrum (Fig. 1f in main text).

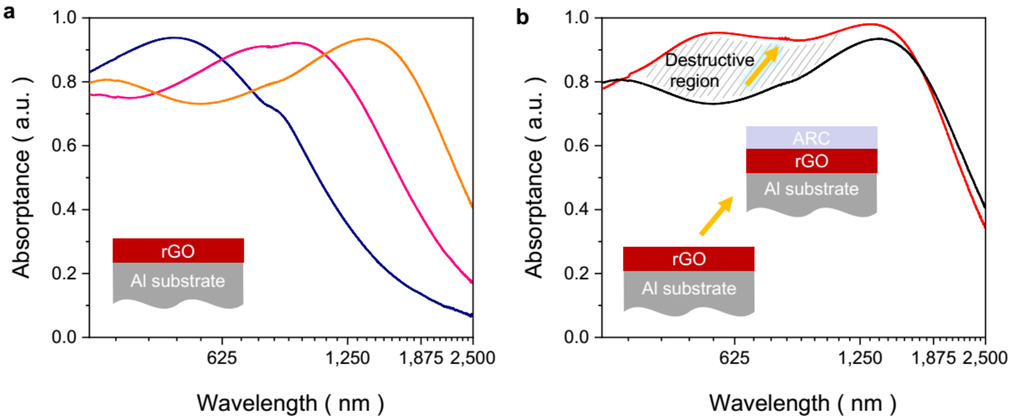

Figure S4. Spectral selectivity of Al|rGO and modification from ARC in Al|rGO|ARC. (a) Spectral absorptance across 300 ~2500 nm of Al|rGOs with rGO thicknesses of 29 (blue line), 53 (pink line) and 88 nm (orange line). All Al|rGOs is thermally reduced at 300 °C from Al|GOs. **(b)** Absorptance enhancement by ARC coating on Al|rGO by modifying the absorption in destructive interference. Black line indicates the

spectral absorptance across 300 ~2500 nm of Al|rGO in which rGO layer is ~88 nm thick. Red line indicates the spectral absorptance of Al|rGO|ARC in which rGO layer is ~100 nm thick and ARC is ~50 nm thick. Destructive region featured with gray lines represents the insufficient absorption band resulting from in destructive interference of single layer of rGO, however, this region is amended because of the modified multilayer interference of rGO|ARC after ARC coating.

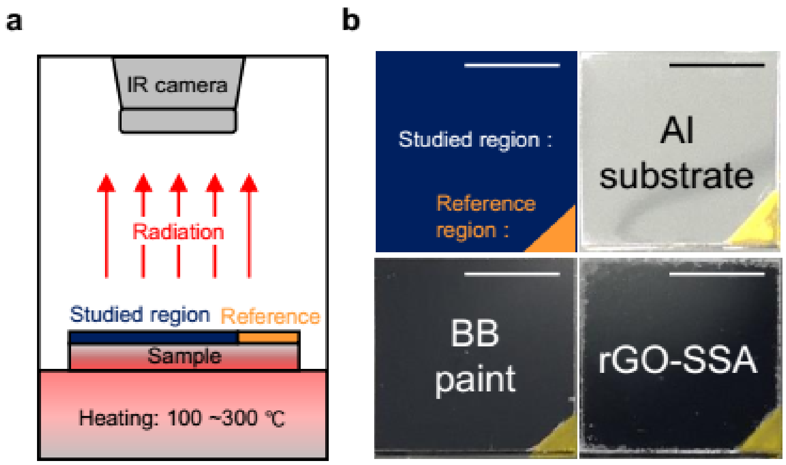

Figure S5. In-situ thermal emittance characterization. (a) Testing setup of in-situ characterization of thermal emittance. Each sample is placed on a polished Al rear (20 x 20 cm) and heated by a hotplate. Al rear is used for blocking the IR emission from the hotplate, thus improving the measuring accuracy. An IR camera is used to capture IR photos of samples in angle of incidence ~8°. Ambient high temperature heat source is prohibited in the testing procedure. **(b)** Scheme of studied region and testing region. For the testing region, sample surface is made coarse for better stick and surface of polyamide tape is also sanded by an abrasive paper (P600). Optical photos of studied samples are also shown with scale bar: 1 cm.

Three typical samples, BB paint, Al substrate and rGO-SSA (Figure S5b), are studied at heating temperature 100~300 ^{o}C. Each sample has a tiny triangular reference region covered by rough PI tape, with already-known emittance ~0.95 adjusted by an embedded thermocouple. In each heating level, apparent temperatures (global emittance ~0.95) of studied region and reference region are recorded in Figure S6 via IR camera. By manually modifying the emittance of studied region until its temperature equals to referenced region,[10] in-situ ε_T of these samples are determined (Fig. 3a in main text).

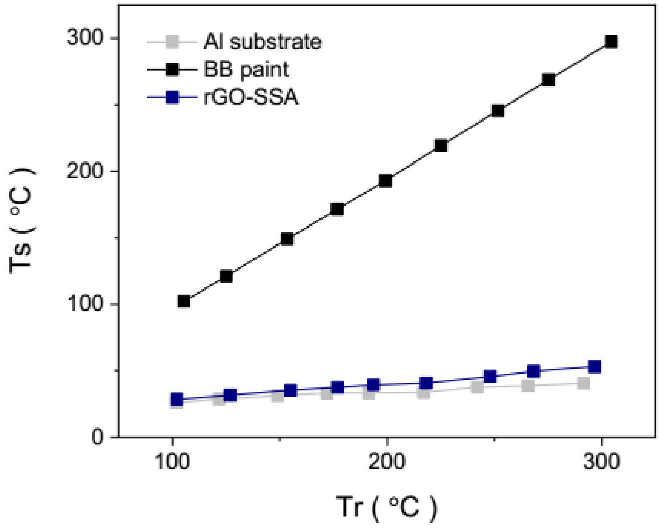

Figure S6. The apparent temperatures of studied region (T_s) and reference region (T_r) of samples measured from in-situ thermal emittance characterization. Gray, black and blue lines indicate Al substrate, BB paint and rGO-SSA respectively. It shows apparent temperature of BB paint (T_s) is almost the same to the reference temperature (T_r, i.e. real temperature), implying BB paint has a near blackbody thermal emittance across 100~300 ^{o}C. While apparent temperature of rGO-SSA is rather closed to bare Al substrate, which implies they have similar thermal emittances (both of them are far smaller than BB paint).

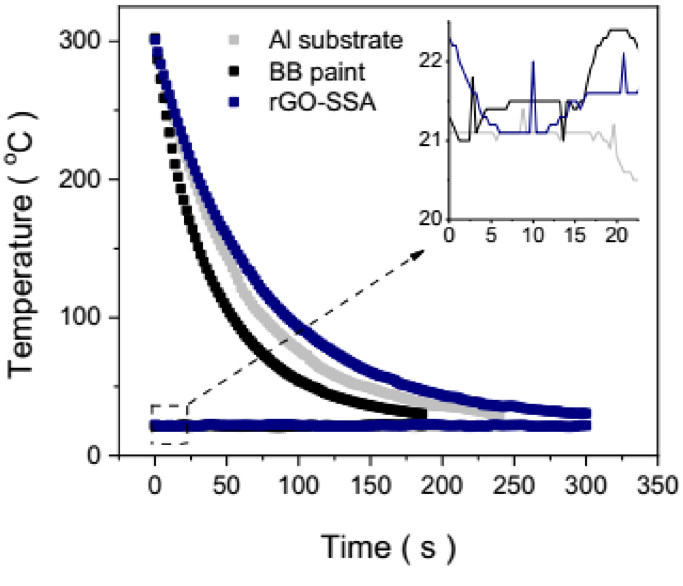

Figure S7. Real-time temperatures of samples and environment recorded during free-cooling test. Gray, black and blue lines indicate Al substrate, BB paint and rGO-SSA respectively. Inset is the enlarged view of ambient temperature at the beginning period. Ambient temperature is stable at 21.5 ±1.0 ^{o}C, and mass of each sample is 0.49 ±0.03 g.

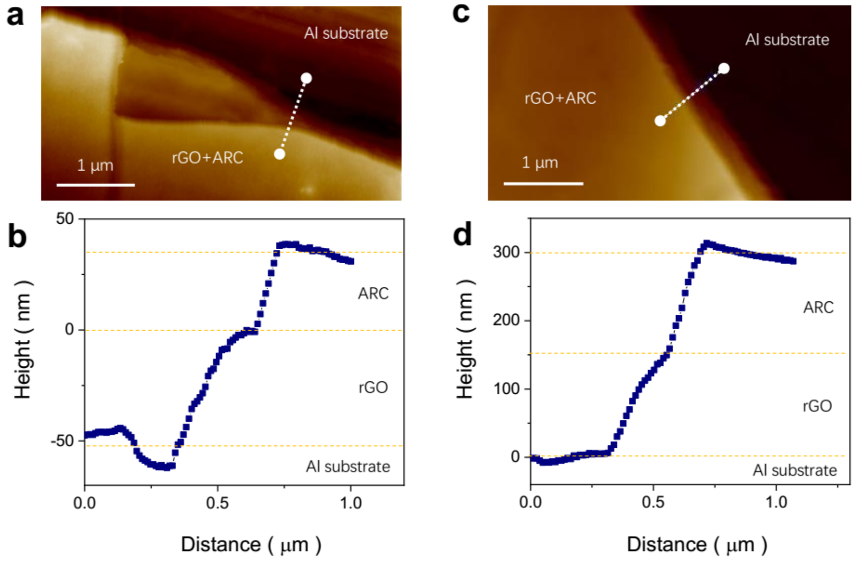

Figure S8. Height profile scanning of the rGO|ARC coatings on Al substrate. (a) and **(b)** Height profile of the cross-section of rGO|ARC coating of Al|rGO|ARC with rGO thickness ~50 nm. **(c)** and **(d)** Height profile of the cross-section of rGO|ARC coating of Al|rGO|ARC with rGO thickness ~150 nm.

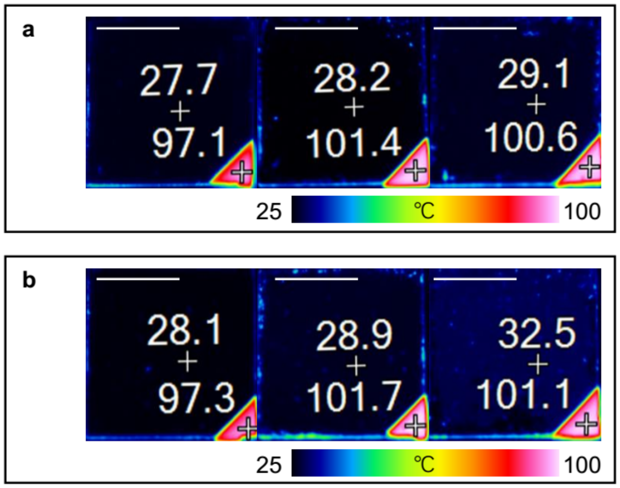

Figure S9. Thermal emittance ε_{100} of rGO-SSAs via IR imaging. (a) IR photos of Al|rGO samples while heating at ~100 ^{o}C with angle of incidence ~8°, cut-off wavelengths of samples are 1.1, 1.8 and 2.4 µm respectively. Scale bar: 1 cm. **(b)** IR photos of Al|rGO|ARC samples while heating at ~100 ^{o}C with angle of incidence ~8°, cut-off wavelengths of samples are 1.3, 2.4 and 3.2 µm respectively. Scale bar: 1 cm.

Table S1. The comparison of absorptance-emittance, cutoff wavelength and thermal stability of rGO-SSAs with the beset achieved of sol-gel SSAs in literature[11].

Sol-gel SSA materials and substrates	Absorptance α-emittance ε	Cutoff wavelength (µm)	Thermal stability	Reference
**Reduced graphene oxide based**				
Aluminum | rGO (1L)	0.74-0.025<br>0.82-0.03<br>0.79-0.04	1.0<br>1.7<br>2.3	--	This work
Aluminum | rGO | ARC (2L)	0.87-0.03<br>0.92-0.04<br>0.89-0.06	1.3<br>2.2<br>3.2	--	
Tungsten | rGO | ARC (2L)	0.94-0.05	3.0	800 °C for 96 h (in argon)	
**Other materials**				
Nickel–alumina cermet on aluminum	0.79-0.04 (1L)<br>0.92-0.05 (2L)<br>0.97-0.05 (3L)	2.1 (1L)<br>2.4 (2L)<br>2.4 (3L)	--	[11]a
Stainless steel | CuO–SiO2 (1L)	0.92-0.2 (80 °C)	4.6	--	[11]b
Stainless steel | Black cobalt | Tin oxide | Nickel (3L)	0.72-0.04	1.2	--	[11]c
Aluminum | CuCoMnOx | SiOx (2L)	0.92-0.04	2.5	100 °C for 2h (in boiling water)	[11]d
Aluminum | CuMnSiOx | CuMnOx | SiO2 (3L)	0.95-0.06	2.1	40 °C for 600 h (condensation)	[11]e

Notes: 1L, 2L, and 3L means the number of coating layer on the substrate is 1, 2, and 3 respectively. ARC is a SiO2 layer in this work. The emittance is sampled at 100 °C without additional caption.

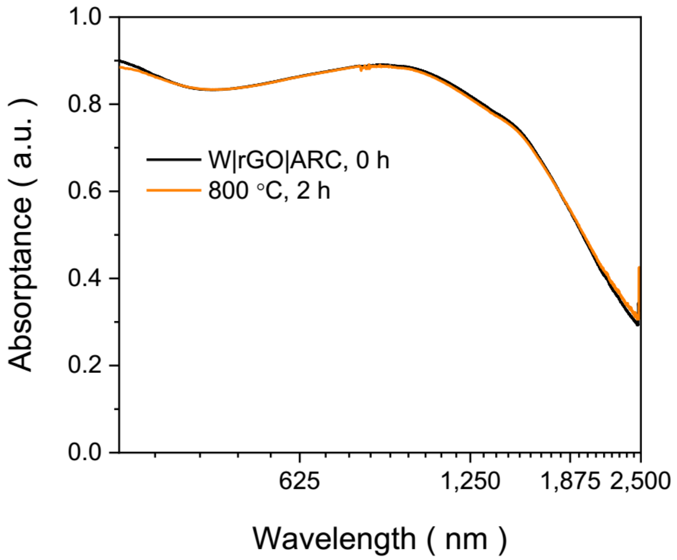

Figure S10. Spectral absorptance of rGO-SSA(W|rGO|ARC) before/after 800 °C for 2 hours. Tungsten substrate (2 x 2 cm sheet) is polished before coating GO|TEOS, and spinning derived W|GO|TEOS is thermal annealed at 800°C under argon protection forming rGO-SSA(W|rGO|ARC). For further long-term test, rGO-SSA is sealed by a silica tube in argon.

Table S2. High temperature stability test for G-SSSA at 800 °C under argon protection.

Samples	Annealing time (h)	Initial *α-ε*	Final *α-ε*	*Δα*	*Δε*	*PC*
Tungsten | rGO | ARC 1	12	0.850-0.05	0.847-0.06	-0.003	0.01	0.008
Tungsten | rGO | ARC 2	96	0.936-0.05	0.925-0.13	-0.011	0.06	0.041

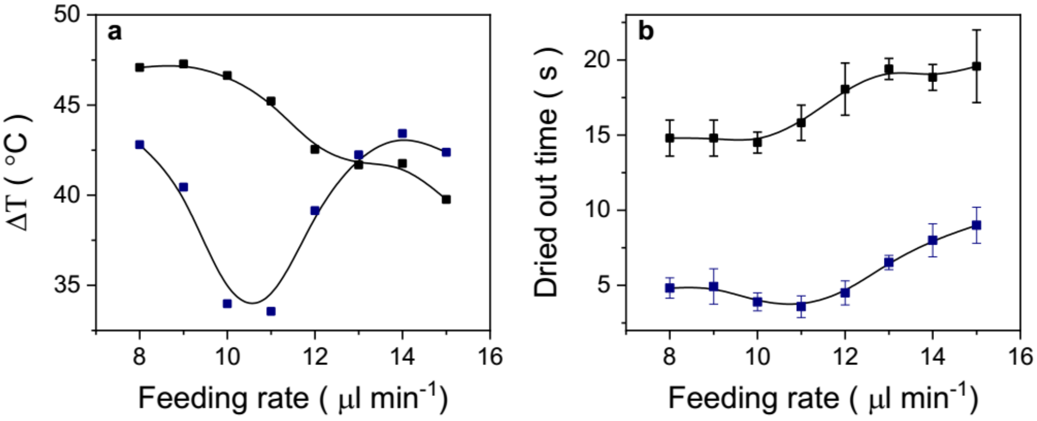

Figure S11 Temperature difference and dried out time information during contact boiling period. (a) The temperature difference (ΔT) and **(b)** dried out time of BB paint absorber (black dotted line) and rGO-SSA (blue dotted line) for different feeding rates (from 8 to 15 μl min^{-1}). The video of whole droplet escaping process is recorded via IR camera. ΔT is read from the reference region temperature of absorber. Dried out time of droplet is judged from the maximum and minimum values of temperature falling procedure (contact boiling period) of absorber (Fig. 5d in main text).

Video S1. Thermal reduction process of rGO-SSA.

In this video, thermal reduction process of rGO-SSA while heating on a hotplate from 50 °C to 300 °C is recorded via digital camera and IR camera. BB paint is pasted on the same substrate of rGO-SSA for vivid comparison between spectrally selective absorption and blackbody-like absorption. The whole video is accelerated by 10 times speed.

Macroscopic appearance change (left panel and the inset on right panel) features the transparent-to-black process of rGO-SSA (drastically happened at ~220 °C), which is significantly different from BB paint and can be easily captured by human eyes, the black

color derived from 300 ^{o}C reduction indicates the high solar absorptance of rGO-SSA. We have also marked the apparent temperatures of rGO-SSA and BB paint on the IR video (right panel). It shows that apparent temperature of BB paint increases from 41.8 ^{o}C to 275.5 ^{o}C, which is nearly the same to the heating temperature. While the apparent temperature of rGO-SSA increases from 27.0 ^{o}C to 61.4 ^{o}C, which is also linearly proportional to the heating temperature but remains in a rather low level, it implies the low thermal emittance nature of rGO-SSA.

References

[1] M. F. Modest, in *Radiative Heat Transfer (Third Edition)*, DOI: https://doi.org/10.1016/B978-0-12-386944-9.50003-0 (Ed: M. F. Modest), Academic Press, Boston **2013**, p. 61.

[2] C. E. Kennedy , National Renewable Energy Laboratory , **2002**.

[3] B. Carlsson, M. Koehl, U. Frei, K. Moeller, in *NASA STI/Recon Technical Report N*, Vol. 95, 1994, 22362.

[4] K. Zhang, L. Hao, M. A. Du, J. Mi, J. N. Wang, J. P. Meng, *Renew. Sust. Energ. Rev.* **2017**, 67, 1282.

[5] S. Brunold, U. Frei, B. Carlsson, K. Möller, M. Köhl, *Sol. Energy* **2000**, 48.

[6] P. N. Dyachenko, S. Molesky, A. Y. Petrov, M. Stormer, T. Krekeler, S. Lang, M. Ritter, Z. Jacob, M. Eich, *Nat. Commun.* **2016**, 7, 11809.

[7] F. Cao, D. Kraemer, L. Tang, Y. Li, A. P. Litvinchuk, J. M. Bao, G. Chen, Z. F. Ren, *Energ. Environ. Sci.* **2015**, 8, 3040.

[8] K. Cui, P. Lemaire, H. Zhao, T. Savas, G. Parsons, A. J. Hart, *Adv. Energy Mater.* **2018**, 8, 1801471.

[9] M. F. Modest, in *Radiative Heat Transfer (Third Edition)*, DOI: https://doi.org/10.1016/B978-0-12-386944-9.50001-7 (Ed: M. F. Modest), Academic

Press, Boston **2013**, p. 1.

[10] L. Xiao, H. Ma, J. Liu, W. Zhao, Y. Jia, Q. Zhao, K. Liu, Y. Wu, Y. Wei, S. Fan, K. Jiang, *Nano Lett.* **2015**, 15, 8365.

[11] a) T. Bostrom, G. Westin, E. Wackelgard, *Sol. Energy Mater. Sol. Cells* **2007**, 91, 38; b) E. Barrera-Calva, J. Méndez-Vivar, M. Ortega-López, L. Huerta-Arcos, J. Morales-Corona, R. Olayo-González, *Research Letters in Materials Science* **2008**, 2008, 1; c) E. Barrera, L. Huerta, S. Muhl, A. Avila, *Sol. Energy Mater. Sol. Cells* **2005**, 88, 179; d) J. Vince, A. Šurca Vuk, U. O. Krašovec, B. Orel, M. Köhl, M. Heck, *Sol. Energy Mater. Sol. Cells* **2003**, 79, 313; e) R. Bayón, G. San Vicente, Á. Morales, *Sol. Energy Mater. Sol. Cells* **2010**, 94, 998.
